# Guillain-Barré Syndrome, Influenza Vaccination, and Antecedent Respiratory and Gastrointestinal Infections: A Case-Centered Analysis in the Vaccine Safety Datalink, 2009–2011

**DOI:** 10.1371/journal.pone.0067185

**Published:** 2013-06-26

**Authors:** Sharon K. Greene, Melisa D. Rett, Claudia Vellozzi, Lingling Li, Martin Kulldorff, S. Michael Marcy, Matthew F. Daley, Edward A. Belongia, Roger Baxter, Bruce H. Fireman, Michael L. Jackson, Saad B. Omer, James D. Nordin, Robert Jin, Eric S. Weintraub, Vinutha Vijayadeva, Grace M. Lee

**Affiliations:** 1 Department of Population Medicine, Harvard Medical School and Harvard Pilgrim Health Care Institute, Boston, Massachusetts, United States of America; 2 Immunization Safety Office, Division of Healthcare Quality Promotion, Centers for Disease Control and Prevention, Atlanta, Georgia, United States of America; 3 Kaiser Permanente Southern California, Pasadena, California, United States of America; 4 Institute for Health Research, Kaiser Permanente Colorado, Denver, Colorado, United States of America; 5 Department of Pediatrics, University of Colorado, Aurora, Colorado, United States of America; 6 Marshfield Clinic Research Foundation, Marshfield, Wisconsin, United States of America; 7 Kaiser Permanente Vaccine Study Center, Oakland, California, United States of America; 8 Group Health Research Institute, Seattle, Washington, United States of America; 9 Center for Health Research-Southeast, Kaiser Permanente Georgia, Atlanta, Georgia, United States of America; 10 HealthPartners Research Foundation, Minneapolis, Minnesota, United States of America; 11 Center for Health Research-Hawaii, Kaiser Permanente Hawaii, Honolulu, Hawaii, United States of America; 12 Division of Infectious Diseases and Department of Laboratory Medicine, Boston Children’s Hospital, Boston, Massachusetts, United States of America; College of Medicine, Hallym University, Republic of Korea

## Abstract

**Background:**

Guillain-Barré Syndrome (GBS) can be triggered by gastrointestinal or respiratory infections, including influenza. During the 2009 influenza A (H1N1) pandemic in the United States, monovalent inactivated influenza vaccine (MIV) availability coincided with high rates of wildtype influenza infections. Several prior studies suggested an elevated GBS risk following MIV, but adjustment for antecedent infection was limited.

**Methods:**

We identified patients enrolled in health plans participating in the Vaccine Safety Datalink and diagnosed with GBS from July 2009 through June 2011. Medical records of GBS cases with 2009–10 MIV, 2010–11 trivalent inactivated influenza vaccine (TIV), and/or a medically-attended respiratory or gastrointestinal infection in the 1 through 141 days prior to GBS diagnosis were reviewed and classified according to Brighton Collaboration criteria for diagnostic certainty. Using a case-centered design, logistic regression models adjusted for patient-level time-varying sources of confounding, including seasonal vaccinations and infections in GBS cases and population-level controls.

**Results:**

Eighteen confirmed GBS cases received vaccination in the 6 weeks preceding onset, among 1.27 million 2009–10 MIV recipients and 2.80 million 2010–11 TIV recipients. Forty-four confirmed GBS cases had infection in the 6 weeks preceding onset, among 3.77 million patients diagnosed with medically-attended infection. The observed-versus-expected odds that 2009–10 MIV/2010–11 TIV was received in the 6 weeks preceding GBS onset was odds ratio = 1.54, 95% confidence interval (CI), 0.59–3.99; risk difference = 0.93 per million doses, 95% CI, −0.71–5.16. The association between GBS and medically-attended infection was: odds ratio = 7.73, 95% CI, 3.60–16.61; risk difference = 11.62 per million infected patients, 95% CI, 4.49–26.94. These findings were consistent in sensitivity analyses using alternative infection definitions and risk intervals for prior vaccination shorter than 6 weeks.

**Conclusions:**

After adjusting for antecedent infections, we found no evidence for an elevated GBS risk following 2009–10 MIV/2010–11 TIV influenza vaccines. However, the association between GBS and antecedent infection was strongly elevated.

## Introduction

Guillain-Barré Syndrome (GBS), the most common cause of acute flaccid paralysis worldwide [1], can be triggered by antecedent gastrointestinal or respiratory infections (including influenza) [2], [3], which are associated with two-thirds of GBS cases [4], [5]. A possible association between GBS and influenza vaccine has been a concern since the 1976 swine-origin influenza vaccination program [6]. Although several studies of subsequent influenza vaccine formulations did not support an elevated GBS risk [7]–[11], monitoring GBS risk following influenza A (H1N1) 2009 monovalent vaccines was considered a public health priority, and multiple surveillance systems were activated [12]. In the Vaccine Safety Datalink (VSD), GBS was significantly associated with monovalent inactivated (MIV) but not seasonal trivalent inactivated (TIV) influenza vaccines in 2009–10, using a self-controlled risk interval design [13], [14] that compared the timing of GBS onset in risk and control intervals following immunization within the same individuals [15]. Although a causal association could not be proven, the findings from this and other surveillance programs [16]–[19] may inform the Countermeasures Injury Compensation Program [20] to include GBS as a potential adverse event following MIV.

In the prior VSD GBS study, five of nine cases with onset in the six weeks following MIV also had an antecedent respiratory infection documented in the medical record within one month prior to GBS onset, compared with one of eight cases following TIV [15]. Of the five GBS cases following MIV with a documented antecedent respiratory infection, three had visited a healthcare provider and been diagnosed with acute upper respiratory infection of multiple or unspecified sites, while the other two patients’ infections had not been medically-attended. The timing of initial MIV availability in VSD coincided with the peak of the second wave of the 2009 influenza A (H1N1) pandemic in late October 2009 [21], [22], while 2009–10 TIV administration mostly preceded this wave [15]. The prior study may have been biased toward a positive GBS/2009–10 MIV association, since some GBS cases soon after vaccination may have been due to influenza virus infection [23]–[25].

Our objectives were to estimate the association between: 1) GBS and receipt of either 2009–10 MIV or 2010–11 TIV (as both vaccine formulations contained the same novel H1N1 antigen), adjusting for patient-level medically-attended infection, and 2) GBS and medically-attended infection, adjusting for 2009–10 MIV/2010–11 TIV receipt.

## Methods

### Study Population

The VSD [26] is a collaboration between the Centers for Disease Control and Prevention (CDC), America’s Health Insurance Plans, and ten health care systems (“sites”). The VSD collects vaccination and medical care data on enrollees, including age, sex, vaccines administered, and International Classification of Diseases, Ninth Revision, Clinical Modification (ICD-9-CM) diagnosis codes for medical encounters in clinic, emergency department, and hospital settings.

Ten sites provided data on over 9 million members: Group Health Cooperative (Washington State); Harvard Vanguard Medical Associates and Harvard Pilgrim Health Care (Massachusetts); HealthPartners Research Foundation (Minnesota); Kaiser Permanente of Colorado; Kaiser Permanente of Georgia; Kaiser Permanente of Hawaii; Kaiser Permanente of Northern California; Kaiser Permanente Northwest (Oregon); Kaiser Permanente of Southern California; and Marshfield Clinic Research Foundation (Wisconsin). Institutional review boards at each site approved this study and determined that the study met the regulatory requirements necessary in order to waive informed consent by the patients for their information to be stored and used for research. A waiver of authorization under the *Standards for Privacy of Individually Identifiable Health Information* (“Privacy Rule”) of the Health Insurance Portability and Accountability Act was authorized.

### Case Finding and Medical Record Review

Potential GBS cases aged ≥6 months were identified using ICD-9-CM diagnosis code 357.0 assigned during clinic, emergency department, or hospital visits from July 2009 through June 2011. Cases were eligible if they were: 1) enrolled at their site for ≥141 days as of the GBS diagnosis, allowing for 2 weeks following the end of the control interval to avoid underascertainment, 2) hospitalized within the month before or after GBS diagnosis, and 3) diagnosed within 1 through 141 days following an eligible vaccination and/or infection, as described below (cases without these exposures were uninformative for the specified analyses and, for efficiency, were not reviewed). Cases were excluded if they had any diagnoses of GBS or chronic inflammatory demyelinating polyneuritis (CIDP) (ICD-9-CM code 357.81) in the prior 5 years within available electronic data.

Eligible cases previously adjudicated for the prior study [15] were included. For newly identified cases, medical records for a minimum of 60 days prior to and following the incident GBS diagnosis were reviewed to confirm cases and to determine GBS onset date. To exclude cases later determined to have CIDP [27] (an exclusionary criterion for GBS diagnosis) [28], additional records were reviewed for patients who had: 1) a CIDP diagnosis anytime following the GBS diagnosis, 2) a possible CIDP diagnosis noted during chart abstractions, and/or 3) a primary inpatient diagnosis for GBS in the 2 weeks through 6 months following the incident GBS diagnosis. Clinician adjudicators at each site applied criteria developed by the Brighton Collaboration [28] to classify GBS and Fisher syndrome (a GBS subtype) into 4 levels of certainty [15].

### Exposure Definitions

We identified 2009–10 MIV and 2010–11 TIV vaccines using electronic vaccination records. An association has not been demonstrated between GBS and 2009–10 TIV or live-attenuated influenza vaccines; these vaccine formulations were thus excluded from analysis for simplicity. We identified medically-attended acute respiratory, gastrointestinal, and unspecified viral infections using ICD-9-CM codes (Table S1) for visits in any setting from July 2009 through June 2011. Codes were adapted from lists used in syndromic surveillance [29]. To avoid recall bias, we excluded from analysis subclinical infections that were recorded only as notes in medical records on or after the GBS diagnosis date, as more recent infections would be more likely to be recorded than less recent infections.

### Study Design

The case-centered method [15], [21], [30]–[33] adjusts for time-varying sources of confounding, including vaccine receipt and infections. To test the hypothesis that there was an excess risk of GBS cases with antecedent vaccination, adjusting for medically-attended infection, logistic regression was used to model the observed-versus-expected odds that vaccination occurred within a biologically plausible period of elevated risk (“risk interval,” 1 through 42 days) prior to GBS onset. The dataset included 1 record for each GBS case with individually matched population data on all vaccinees as of the GBS onset date of the same site, age group, sex, and medically-attended infection status (yes/no in the 1 through 42 days prior to GBS onset). (Note that only those infections that were medically-attended could be identified in the individually matched population data, as it would have been infeasible to conduct medical record reviews or interviews to identify subclinical infections in non-GBS cases.) The model included 2 variables: a binary indicator of the outcome (whether vaccination was inside or outside of the risk interval) and the log of the “expected” odds of being in the risk interval, specified as an offset. The “expected” odds were derived from the proportion of enrolled vaccinees similar to the GBS case among the whole population who were still in a post-vaccination risk interval for their most recent dose (1 or 2) on the onset date of the GBS case. The intercept yielded the odds ratio estimate for vaccination in the risk interval prior to GBS onset vs. in the control interval, adjusting for site, age group, sex, and infection status. Risk differences were calculated using the formula: (odds ratio –1)*p_0_, where *p_0_* was the GBS background rate of 1.5 per 100,000 person-years [5], [34] (assumed to be known without error), scaled to a 6-week risk interval.

### Primary Analysis

Vaccinations were considered to be in the risk interval if they were administered in the 1 through 42 days (i.e., 1 through 6 weeks) prior to GBS onset, consistent with biological plausibility and prior studies [6], [15], 21. Vaccinations in the prior 43 through 49 days were excluded as a washout interval, to allow for the possibility of the risk period extending up to an additional week [19]. Vaccinations in the prior 50 through 126 days (i.e., 8 through 18 weeks) were in the control interval, consistent with the definition used in the prior study [15]. Cases were considered to have antecedent infection if they had a medically-attended respiratory, gastrointestinal, or unspecified viral infection (Table S1) in the 1 through 42 days prior to GBS onset.

An analogous strategy was used to test the second hypothesis regarding an excess risk of GBS cases with antecedent infection, adjusting for vaccination. That is, medically-attended respiratory, gastrointestinal, or unspecified viral infections were considered to be in the risk interval if they were in the 1 through 42 days prior to GBS onset. Infections in the prior 43 through 49 days were excluded. If the patient’s most recent medically-attended infection was in the 50 through 126 days prior to GBS onset, then the infection was considered to be in the control interval. Cases were considered to have antecedent vaccination if they received a dose of 2009–10 MIV or 2010–11 TIV in the 1 through 42 days prior to GBS onset. Analyses were conducted using SAS, version 9 (SAS Institute Inc., Cary, North Carolina).

### Secondary Analyses

Secondary analyses were defined a priori to assess the magnitude of effect for explicitly adjusting for infection and vaccination, and to test the sensitivity of the primary analysis results to alternative infection and risk interval definitions [35]. For the GBS/vaccination model, we removed adjustment for infection by redefining the stratum for each GBS case, including onset date, age group, sex, and site. This analysis differed from a secondary analysis in the prior VSD GBS study [15] by including an additional vaccination season (2010–11) to improve power, by using in the offset term the most recent dose (1 or 2) prior to the GBS onset date rather than only the first dose, and by imposing an enrollment criterion for all vaccinees, similar to the vaccinated GBS cases.

All confirmed cases (Brighton Criteria Levels 1–4, where Level 4 is “a reported event of GBS or Fisher Syndrome with insufficient evidence to meet the case definition”) [28] were included in all analyses, except for a secondary analysis to assess the effect of restricting to Brighton Criteria Levels 1–3. To assess effect modification by vaccine type, estimates for 2009–10 MIV and for 2010–11 TIV were also separately calculated.

The infection definition was restricted to respiratory infection-only in four secondary analyses. Two definitions were ICD-9-CM code based: 1) upper or lower respiratory tract infection diagnosis (Table S1), and 2) influenza diagnosis specifically. Other definitions required a respiratory tract infection diagnosis and on the same day 3) fever [36], or influenza or respiratory syncytial virus (RSV) laboratory test *ordered* (a potential marker for acute infection, regardless of test result), and 4) fever, or *positive* influenza or RSV laboratory test. Two additional analyses focused on gastrointestinal infection: 1) gastrointestinal infection diagnosis (Table S1), and 2) expanding 1) to also include a diarrhea diagnosis (787.91), which is nonspecific for infectious diarrhea.

Although a 6-week risk interval is standard for this research question, the period of greatest risk elevation may be shorter [10], [37]. As vaccination timing was precisely known [38], two shorter vaccine risk intervals (4-weeks and 3-weeks) were selected, and the 6-week interval was also subdivided into 3 intervals (i.e., days 1 through 7, 8 though 28, and 29 through 42). Analogous secondary analyses for shorter risk intervals following infection would not be valid, as the timing of infection onset was not known to this level of precision.

## Results

### Vaccination and Infection Patterns

The total number of vaccinations administered was 1,267,745 2009–10 MIV first doses and 2,798,788 2010–11 TIV first doses. The number of patients with medically-attended infection was 3,770,362, including 182,434 influenza diagnoses, 3,014,506 other respiratory infections, 438,444 unspecified viral infections, and 134,978 gastrointestinal infections.

**Figure 1:**
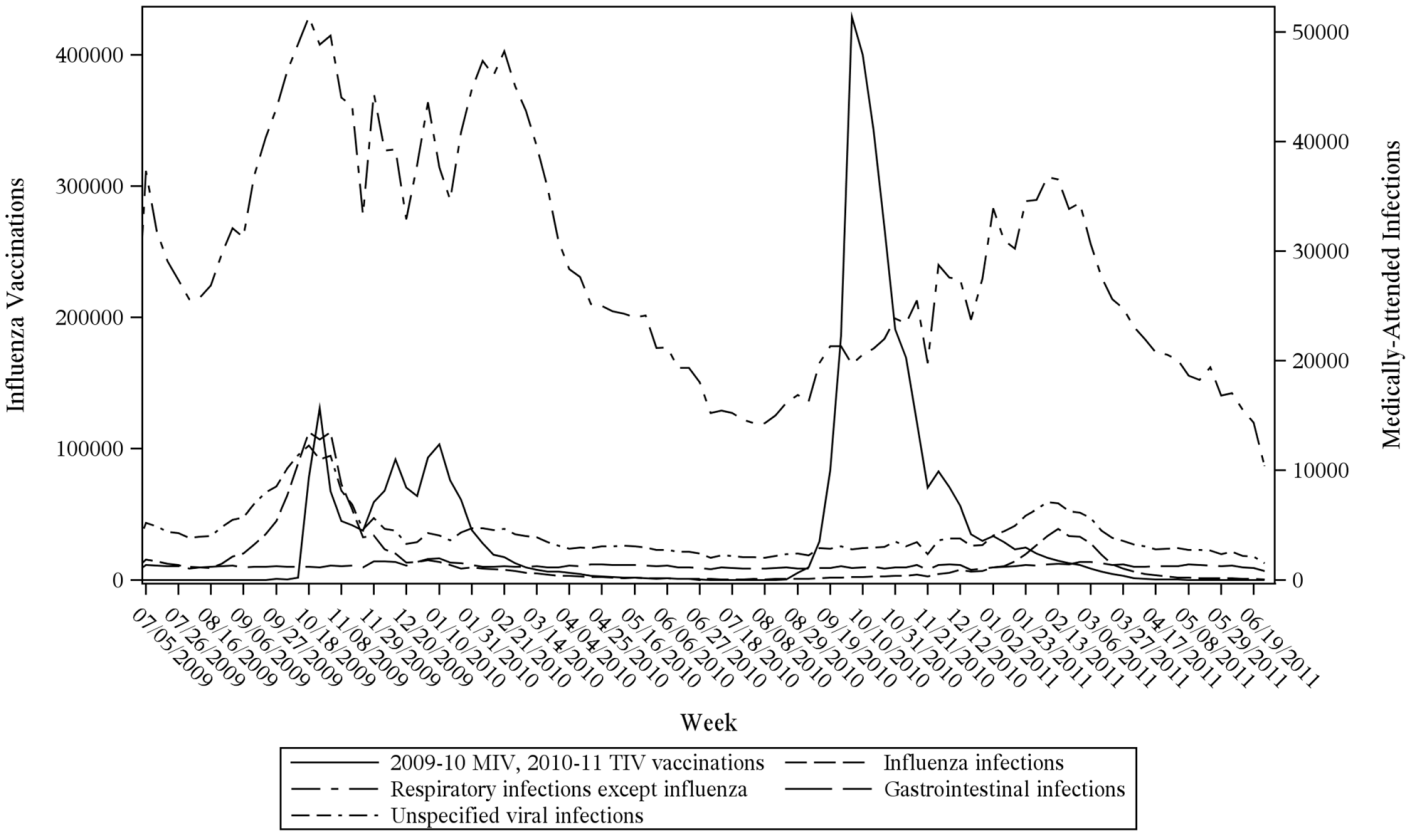
Influenza vaccinations and medically-attended infections, Vaccine Safety Datalink, July 2009–June 2011.

**Figure 2:**
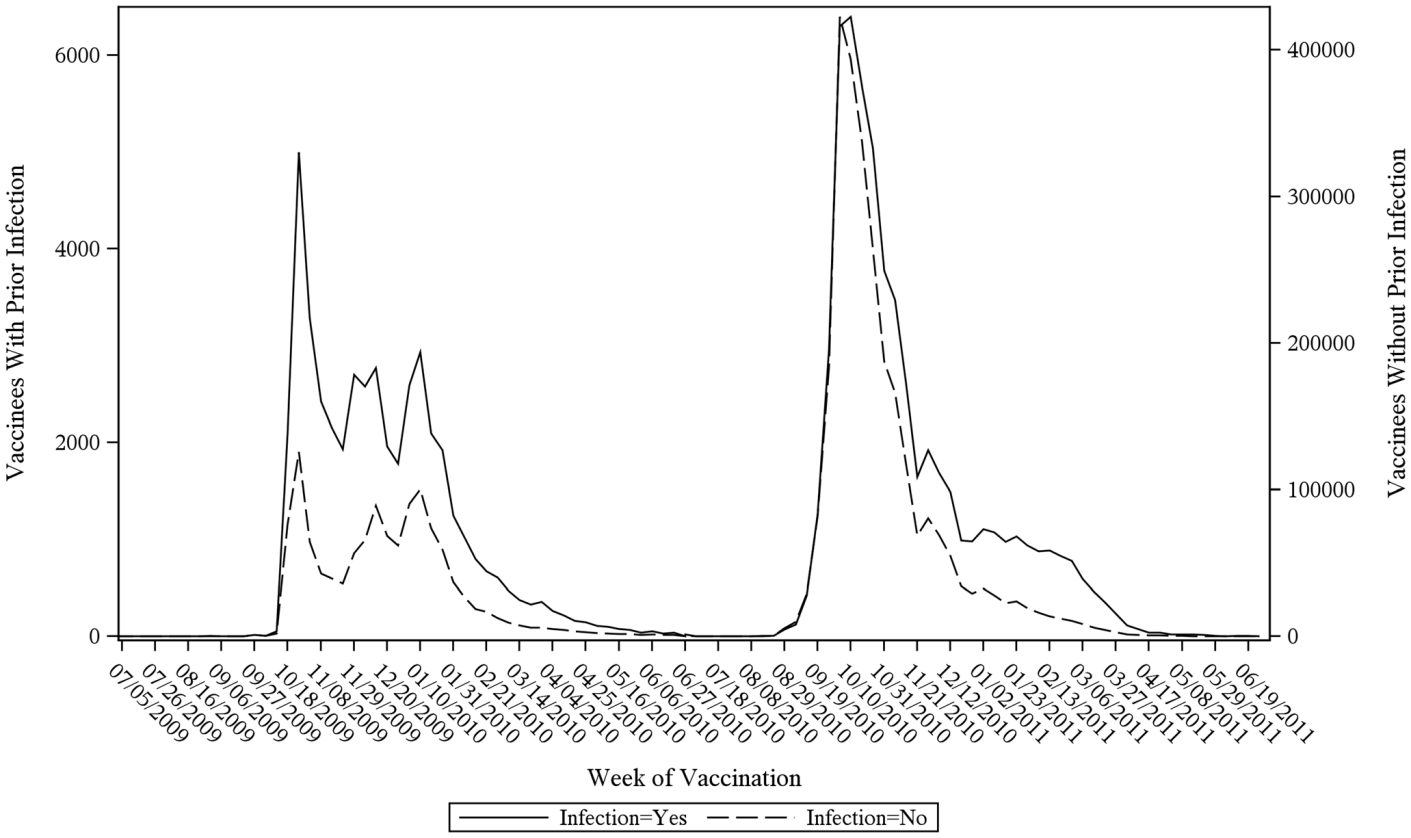
Weekly administration of influenza vaccines, by medically-attended infection status. Weekly administration of 2009–10 monovalent inactivated influenza vaccine and 2010–11 trivalent inactivated influenza vaccine, by medically-attended infection status in prior 6 weeks (yes/no).

2009–10 MIV administration began October 2009, concurrent with peaks of medically-attended infections for influenza, other respiratory tract infections, and unspecified viral infections (Figure 1). In contrast, 2010–11 TIV administration peaked in October 2010 and was mostly complete prior to the February 2011 peak of seasonal infections (Figure 1). Vaccination timing was similar between vaccinees who were vs. were not recently infected (Figure 2), suggesting that adjusting for infection in case-centered analysis would have a modest effect.

### GBS Case Confirmation and Exposures

The number of eligible GBS cases in the electronic data was 469; of these, 179 (38%) had an eligible vaccination or infection exposure in the 1 through 141 days prior to GBS diagnosis. Seventy-two cases in the electronic data had an antecedent vaccination, and 133 cases had an antecedent infection (Table 1); 26 cases had both exposures. Of the 153 exposed cases for whom medical records were available for review and who did not have GBS onset prior to exposure, 78 were confirmed as GBS or Fisher Syndrome Level 1–4, for an overall positive predictive value of 51.0%. The most common reason why cases were not confirmed was an alternative diagnosis (Table 1). Of confirmed cases, 44 (56%) were male, 42 (54%) were ≥50 years-old (Table S2), and the median length between GBS onset and diagnosis was 5 days (interquartile range: 2, 11).

**Table 1:** Disposition of eligible patients with Guillain-Barré Syndrome (GBS), by vaccination and infection exposure status^a^ and timing of diagnosis, Vaccine Safety Datalink, 2009–2011.

		GBS cases with influenza vaccination^b^ in 1–141 days prior to GBS diagnosis (n = 72)		GBS cases with infection^c^ in 1–141 days prior to GBS diagnosis (n = 133)	
Category	Level	1–42 days (n, %) (N = 32)	43–141 days (n, %) (N = 40)	1–42 days (n, %) (N = 97)	43–141 days (n, %) (N = 36)
Eligible and confirmed (Brighton Collaboration Criteria)	GBS Level 1	3 (9)	1 (3)	9 (9)	1 (3)
	GBS Level 2	6 (19)	10 (25)	18 (19)	6 (17)
	GBS Level 3	2 (6)	1 (3)	2 (2)	1 (3)
	GBS Level 4	3 (9)	5 (13)	8 (8)	4 (11)
	Fisher Syndrome Level 1	0	3 (8)	1 (1)	0
	Fisher Syndrome Level 2	1 (3)	1 (3)	4 (4)	0
	Fisher Syndrome Level 3	0	0	1 (1)	0
	Fisher Syndrome Level 4	0	1 (3)	1 (1)	0
Medical records unavailable for review		1 (3)	2 (5)	4 (4)	2 (6)
GBS onset prior to exposure		2 (6)	0	17 (18)	0
Not confirmed as GBS	Chronic inflammatory demyelinating polyneuritis (CIDP)^d^	0	1 (3)	5 (5)	5 (14)
	Alternative diagnosis other than CIDP^e^	9 (28)	9 (23)	16 (16)	7 (19)
	No documentation of GBS in medicalrecord	3 (9)	5 (13)	10 (10)	6 (17)
	Remote GBS occurrence listed inmedical history	1 (3)	1 (3)	1 (1)	3 (8)
	Follow-up care for prior GBS diagnosis	0	0	0	1 (3)
	Coding error	1 (3)	0	0	0
Positive predictive value among patients with available medical records and who did not have GBS onset prior to exposure		15/29 = 51.7%	22/38 = 57.9%	44/76 = 57.9%	12/34 = 35.3%

a 26 cases had both prior exposures in the 1 through 141 days prior to GBS diagnosis.

b 2009–10 monovalent inactivated influenza vaccine or 2010–11 trivalent inactivated influenza vaccine.

c Medically-attended respiratory, gastrointestinal, or unspecified viral infection.

d For cases with electronic diagnoses of CIDP (n = 9): median length between GBS diagnosis and first CIDP diagnosis: 51 days (interquartile range: 4, 81).

e Alternative diagnoses included: transverse myelitis/myelitis (including post-viral or varicella zoster virus), acute disseminated encephalomyelitis, conversion disorder/functional component, viral illness, viral ataxia, viral myopathy, Charcot-Marie-Tooth disease, diabetic amyotrophy or myopathy, generalized vasculitis, vasculitis neuropathy, hereditary brachial neuropathy, Lyme meningitis, multiple cranial neuropathy, multiple sclerosis, sarcoid neuropathy, encephalomyopathy radiculitis, myasthenia gravis, steroid myopathy, paresis, polymyositis, and not specified.

The intersection of vaccination and medically-attended infection exposures in risk and control intervals prior to GBS onset is in Table S3. In the 6 weeks prior to GBS onset, 18 cases received vaccine and 44 cases had medically-attended infection. Three cases had both exposures in the risk interval; all 3 were in the 2009–10 influenza season, and all were diagnosed with ICD-9-CM 465.9 (acute upper respiratory infection of multiple or unspecified sites). An additional 4 cases had respiratory symptoms (including upper respiratory infection, cold, and bronchitis) noted in the medical records in the risk interval prior to GBS onset, but these infections were not medically-attended. Thus, among 18 patients with vaccination in the risk interval prior to GBS onset, 7 (39%) had symptoms of a respiratory infection documented in the medical record, 3 of which were medically-attended.

Fifteen cases had both a medically-attended infection shortly (in the 1 through 4 days) prior to GBS onset and also infectious symptom details noted in medical records. The median length between infection onset and most recent medically-attended infection diagnosis was 13 days (range: 7–93). Symptoms corresponded with infection diagnoses, e.g., a patient with cough, runny nose, headache, wheezing, and shortness of breath was diagnosed with bronchitis. Recorded infection symptoms were not neurologic in nature (e.g., weakness, tingling), suggesting that prodromal GBS was not misdiagnosed as acute infection. The most commonly diagnosed infections were acute upper respiratory infection, pneumonia, and bronchitis (Table 2).

**Table 2:** Most recent medically-attended infection diagnoses within 1 through 42 days prior to confirmed Guillain-Barré syndrome (GBS) onset, by recent influenza vaccination^a^ status, Vaccine Safety Datalink, 2009–2011.

Infection type	ICD-9-CM Code	Description	Patients with infection and influenza vaccination 1 through 42 days prior to GBS onset (n = 3)	Patients with infection but no influenza vaccination 1 through 42 days prior to GBS onset (n = 41)^b^
Upper or lowerrespiratory tract	382.9	Unspecified otitis media	–	5
	460	Acute nasopharyngitis	–	1
	461.9	Acute sinusitis unspecified	–	2
	462	Acute pharyngitis	–	3
	463	Acute tonsillitis	–	2
	464.00	Acute laryngitis and tracheitis without obstruction	–	1
	465.9	Acute upper respiratory infections of unspecified site	3	6
	466.0	Acute bronchitis	–	4
	482.42	Methicillin resistant pneumonia due to *Staphylococcus aureus*	–	1
	486	Pneumonia, organism unspecified	–	7
	487.1	Influenza with other respiratory manifestations	–	2
	490	Bronchitis not specified as acute or chronic	–	7
	510.9	Empyema without fistula	–	2
	513.0	Abscess of lung	–	1
Gastrointestinal	008.45	Intestinal infection due to *Clostridium difficile*	–	1
	008.8	Intestinal infection due to other organism not elsewhere classified	–	1
	009.0	Infectious colitis enteritis and gastroenteritis	–	1
	009.2^c^	Infectious diarrhea	–	1
Unspecifiedviral infection	079.99	Unspecified viral infection	–	2

a 2009–10 monovalent inactivated influenza vaccine or 2010–11 trivalent inactivated influenza vaccine.

b There may be >1 medically-attended infection diagnosis on the same day, resulting in more infection diagnoses than GBS patients.

c The patient with a diagnosis of infectious diarrhea also tested positive for *Campylobacter jejuni*.

### Primary Analysis

The GBS/vaccination association, adjusting for medically-attended antecedent infection, was 1.54 (95% confidence interval [CI], 0.59–3.99). In contrast, the GBS/infection association, adjusting for antecedent vaccination, was much stronger (odds ratio = 7.73, 95% CI, 3.60–16.61) (Table 3, analysis 1). Assuming a GBS background rate of 1.5 per 100,000 person-years [5], [34], the risk difference for vaccination was 0.93 GBS cases per million doses (95% CI, −0.71–5.16), and for medically-attended infection was 11.62 GBS cases per million infected patients (95% CI, 4.49–26.94).

**Table 3:** Case-centered analysis results of Guillain-Barré syndrome (GBS) with prior vaccination and with prior infection, Vaccine Safety Datalink, 2009–2011.

							GBS with prior influenza vaccination^a^			GBS with prior medically-attended infection^b^		
Analysis Number		Brighton Collaboration Criteria Levels for GBS case inclusion		Risk interval (inclusive) for exposure prior to GBS onset	Control interval (inclusive) for exposure prior to GBS onset	Medically-attended infection definition^c^	GBS cases exposed in risk interval	GBS cases exposed in control interval	Odds ratio (95% CI)	GBS cases exposed in riskinterval	GBS cases exposed in control interval	Odds ratio(95% CI)
Primary analysis												
1	1–4		1–42		50–126	A	17	18	1.54 (0.59–3.99)	43	8	7.73 (3.60–16.61)
For GBS with prior influenza vaccination, no adjustment for infection status. For GBS with prior infection, no adjustment for influenza vaccination status												
2	1–4		1–42		50–126	A	17	18	1.64 (0.62–4.28)	43	8	7.93 (3.69–17.01)
Restrict to Brighton Level 1–3 cases												
3	1–3		1–42		50–126	A	13	14	1.27 (0.42–3.83)	34	5	10.38 (4.02–26.81)
Restrict to cases with antecedent 2009–10 monovalent inactivated influenza vaccine												
4	1–4		1–42		50–126	A	8^d^	5	1.97 (0.51–7.61)^e^	Not applicable		
Restrict to cases with antecedent 2010–11 seasonal trivalent inactivated influenza vaccine												
5	1–4		1–42		50–126	A	9	13	1.19 (0.29–4.94)^f^	Not applicable		
Alternative infection definitions												
6	1–4		1–42		50–126	B	17	18	1.60 (0.61–4.15)	38	6	8.87 (3.71–21.20)
7	1–4		1–42		50–126	C	17	18	1.64 (0.62–4.29)	2	0	N/A
8	1–4		1–42		50–126	D	17	18	1.63 (0.62–4.28)	4	0	N/A
9	1–4		1–42		50–126	E	17	18	1.63 (0.62–4.28)	4	0	N/A
10	1–4		1–42		50–126	F	17	18	1.64 (0.62–4.29)	4	0	N/A
11	1–4		1–42		50–126	G	17	18	1.55 (0.59–4.05)	6	2	4.11 (0.83–20.43)
Alternative risk intervals												
12	1–4		1–28		50–126	A	10	18	1.23 (0.38–4.01)			
13	1–4		1–21		50–126	A	8	17^g^	1.29 (0.35–4.72)	Not reported: timing of infection onset was		
14	1–4		1–7		50–126	A	2	17	0.64 (0.09–4.33)	not known to this level of precision		
15	1–4		8–28		50–126	A	8	18	1.32 (0.37–4.70)			
16	1–4		29–42		50–126	A	7	18	1.44 (0.39–5.26)			

a Adjusted for GBS onset date, age group, sex, site, and medically-attended infection status in 1 through 42 days prior to GBS onset.

b Adjusted for GBS onset date, age group, sex, site, and influenza vaccination status (2009–10 monovalent inactivated influenza vaccine or 2010–11 trivalent inactivated influenza vaccine) in 1 through 42 days prior to GBS onset.

c Medically-attended infection definitions: A. Respiratory tract, acute gastrointestinal, or unspecified viral infection; B. Respiratory tract including influenza; C. Influenza; D. Respiratory tract with fever and/or influenza or respiratory syncytial virus lab test *ordered* on same day; E. Respiratory tract with fever and/or *positive* influenza or respiratory syncytial virus lab test on same day; F. Acute gastrointestinal; G. Acute gastrointestinal and/or diarrhea.

d The prior VSD GBS study reported 9 cases in the risk interval and 4 cases in the control interval following 2009–10 MIV [15]. One vaccinated GBS case in the risk interval was uninformative in case-centered analysis (100% probability of being in risk interval), so 8 are reported here. There are 5 cases in the control interval for this study because 1 case was dropped (onset occurred within the newly established 43 through 49 day washout interval) while 2 cases were gained (1 case from an additional VSD site added for this study, and 1 case newly identified at a VSD site included in the prior study).

e A post-hoc analysis restricting to cases with antecedent 2009–10 monovalent inactivated influenza vaccine and no adjustment for infection status yielded a point estimate slightly further from the null (odds ratio = 2.10, 95% CI: 0.54–8.22).

f A post-hoc analysis restricting to cases with antecedent 2010–11 seasonal trivalent inactivated influenza vaccine and no adjustment for infection status yielded a point estimate slightly further from the null (odds ratio = 1.25, 95% CI: 0.30–5.29).

g Although analysis 13 had the same control interval definition as analysis 12 (days 50–126), analysis 13 had one fewer case in the control interval (17 vs. 18). This is because cases are only informative if they have a nonzero probability of being in either the risk or control intervals. The dropped case occurred in April 2011, with a 0.8% probability of being exposed in the 1 through 42 day risk interval in analysis 12, but a 0% probability of being exposed in the 1 through 21 day risk interval in analysis 13.

### Secondary Analyses

For the GBS/vaccination association, removing explicit adjustment for antecedent infection moved the point estimate modestly further from the null, from 1.54 to 1.64 (Table 3, analysis 2). The effect on the GBS/infection association of removing explicit adjustment for antecedent vaccination was similar, from odds ratio = 7.73 to 7.93.

Restricting to Brighton Criteria Levels 1–3 did not meaningfully impact the GBS/vaccination association, yet further strengthened the GBS/infection association to odds ratio = 10.38 (analysis 3). The point estimates for the GBS/vaccination association appeared higher for 2009–10 MIV than for 2010–11 TIV, but were not significantly different (analyses 4–5). Of the 22 GBS cases following 2010–11 TIV vaccination, 8 (36%) previously received 2009–10 MIV.

The GBS/vaccination association did not become elevated under any alternative infection or risk interval definitions (analyses 6–16). The GBS/infection association strengthened to odds ratio = 8.87 upon restriction to respiratory tract infection as the most recent infection type (analysis 6).

## Discussion

In the VSD population, there was no statistically significant association between GBS and 2009–10 MIV and 2010–11 TIV combined (odds ratio = 1.54, 95% CI, 0.59–3.99; risk difference = 0.93 per million doses, 95% CI, −0.71–5.16). This finding was robust to alternative infection and risk interval definitions. In contrast, GBS was strongly associated with infection (odds ratio = 7.73, 95% CI, 3.60–16.61; risk difference = 11.62 per million infected patients, 95% CI, 4.49–26.94), especially respiratory infection, consistent with prior studies [10], [39], [40].

Prior observational studies assessing the GBS/2009–10 MIV association have important methodological limitations regarding antecedent infection adjustment (Table S4). Passive surveillance studies [41] had unreliable information on relevant co-exposures, including infections. Case-control studies [40], [42] may be subject to misclassification bias, with potentially differential infection ascertainment between cases and controls. In addition, exposures ascertained from medical records [15]–[18], [43], [44] were recorded *after* GBS onset and can thus be biased; records for patients with one possible documented cause of GBS (e.g., vaccination) may have under-ascertainment of alternative exposures (e.g., infection). Furthermore, several studies [15]–[18], [43]–[45] systematically ascertained antecedent infections during risk but not during control periods, so analyses were not designed to formally adjust for infection. Studies using a self-controlled risk interval design [15], [16], [18] were limited because 2009–10 MIV administration and wild-type infections coincided, such that some cases in the risk interval following vaccination may have been due to infection. The direction of this bias changed the following season, since 2010–11 TIV administration preceded infections (Figure 1), and some cases in the *control* interval for vaccination may have been due to infection.

In contrast, this study used the case-centered design. This design fully adjusted for seasonal exposures such as infections, including the coincidental timing of vaccination and infection in 2009–10 and the different relative timing of vaccination and infection in 2010–11. This design had been used in a secondary analysis in the prior VSD GBS study using data from 2009–10 (odds ratio = 2.0, 95% CI, 0.5–8.1) [15]. The results of the current study, using data from both 2009–10 and 2010–11, are consistent with that analysis. In addition, explicit adjustment within the case-centered design for patient-level medically-attended infections (yes/no in the 1 through 42 days prior to GBS onset) did not substantially change the results (Table 3, analyses 1 vs. 2). This does not suggest that infection is not an important confounder of the association between vaccination and GBS, but rather that infection was not associated with the *timing* of vaccination. Such an adjustment may be more important in future influenza seasons, if recently infected and uninfected vaccinees receive the timing of their vaccine differentially (e.g., due to the MIV and TIV precaution of moderate to severe acute illness with or without fever) [46]. By restricting adjustment to infections that were medically-attended, all informative infections were recorded prior to GBS onset, and patient- and population-level (control) infection data were ascertained consistently.

The GBS/vaccine association point estimates appeared slightly higher in the 2009–10 season than in the 2010–11 season, although they were not significantly different (Table 3). One explanation for a possibly higher risk in 2009–10 is that the population did not have prior exposure to wild-type 2009 H1N1 infection and may have had a more robust immune response to the vaccine containing the novel antigen. However, chance may be a more likely explanation, as more than a third of cases following 2010–11 TIV had prior 2009–10 MIV exposure, yet did not have GBS in 2009–10.

Our study has at least five potential limitations. First, some infection and vaccine exposures may not have been identified using electronic medical records, reducing power. Subclinical infections for which healthcare was not sought and vaccinations administered in nontraditional settings may have been missed [47], [48]. However, missing infections and vaccinations would not bias analyses, as long as exposures were equally likely to be missed in risk as in control intervals. Second, there may be uncontrolled confounding. In the case-centered design, a confounder would be a factor associated with both the outcome of GBS and also the exposure, which is the *timing* of vaccination. For example, within a given stratum defined by people of the same site, age group, sex, and infection status, there could be uncontrolled confounding if a factor associated with increased GBS risk were also associated with receiving vaccination earlier or later than the other people within that same stratum. We are not aware of an example of such a potential confounder, but one or more may theoretically exist. As with the self-controlled risk interval design, the case-centered design restricted analyses to vaccinees only, thus removing any confounders related to the propensity to receive vaccination at all. Third, the case-centered design restricting to vaccinees has reduced power compared with the self-controlled risk interval design. This is because all cases with GBS onset in the risk or control interval are equally informative in the self-controlled risk interval design, while any cases very early in (or after) the vaccination season have nearly 100% (or 0%) probability of being in the risk interval and are thus uninformative or minimally informative in a case-centered design restricting to vaccinees. Future analyses using the case-centered design could improve power (at the potential expense of increasing bias) by defining the expected odds of being in the risk interval differently for such otherwise uninformative cases, e.g., by including individuals who were unvaccinated but otherwise similar in risk to the early vaccinees. Fourth, only the most recent 2009–10 MIV dose, 2010–11 TIV dose, and medically-attended infections prior to GBS onset were assessed. For simplicity, multiple vaccine doses or infection visits per patient were not considered, nor were all possibly relevant vaccine formulations and wildtype infections (e.g., cytomegalovirus or Epstein-Barr virus) [2], [3]. Fifth, the Brighton Collaboration criteria were developed neither to classify cases into subtypes (e.g., demyelinating or axonal [1]), nor to capture all clinical syndromes in the GBS spectrum [28]. Consequently, we may have under-ascertained atypical GBS case-patients who did not meet these classification criteria, e.g., due to lack of flaccid paralysis [49]. However, the incidence of such cases is very low [1], [28], and under-ascertainment, if similar in risk and control intervals, was unlikely to bias reported estimates.

In conclusion, this study found no evidence for a GBS/2009–10 MIV/2010–11 TIV influenza vaccines association, excluding a risk greater than 5.2 cases per million doses, yet strong evidence for a GBS/infection association. This should inform evaluations of the relative benefits and harms of influenza vaccine, especially in light of the risks of natural influenza infection, including GBS. As influenza vaccines are administered seasonally and a high proportion of GBS cases are associated with seasonal infections, future evaluations should consider methodologies that adjust for patient-level effects of seasonal exposures.

## Supporting Information

Table S1 **International Classification of Diseases, Ninth Revision, Clinical Modification (ICD-9-CM) diagnosis codes for defining medically-attended acute infections.**
(DOCX)

Table S2 **Characteristics of confirmed Guillain-Barré syndrome (GBS) cases according to prior influenza vaccination and infection exposure status, Vaccine Safety Datalink, 2009–2011.**
(DOCX)

Table S3 **Numbers of patients with confirmed Guillain-Barré syndrome (GBS) by vaccination and infection exposure status and observation interval, Vaccine Safety Datalink, 2009–2011.**
(DOCX)

Table S4 **Ascertainment of and adjustment for antecedent infection in selected prior observational studies of 2009–10 pandemic H1N1 vaccination and Guillain-Barré syndrome.**
(DOCX)
