# Supplementary material for: Guillain-Barré Syndrome, Influenza Vaccination, and Antecedent Respiratory and Gastrointestinal Infections: A Case-Centered Analysis in the Vaccine Safety Datalink, 2009–2011
Source: PLoS One. 2013 Jun 26;8(6):e67185. doi: 10.1371/journal.pone.0067185 (PMC3694016; doi:10.1371/journal.pone.0067185)
Supplement: Table S1 — International Classification of Diseases, Ninth Revision, Clinical Modification (ICD-9-CM) diagnosis codes for defining medically-attended acute infections. (DOCX) [file pone.0067185.s001.docx]

**Table S1. International Classification of Diseases, Ninth Revision, Clinical Modification (ICD-9-CM) diagnosis codes for defining medically-attended acute infections.**

| **Infection type** | **ICD-9-CM codes (subcodes included)** | **Description** |
| --- | --- | --- |
| Upper or lower respiratory tract | 032 | Diphtheria |
|  | 033 | Whooping cough |
|  | 034 | Streptococcal sore throat and scarlet fever |
|  | 052.1 | Varicella with pneumonia |
|  | 055.1 | Postmeasles pneumonia |
|  | 055.2 | Postmeasles otitis media |
|  | 073 | Ornithosis |
|  | 079.3 | Rhinovirus infection not otherwise specified |
|  | 079.6 | Respiratory syncytial virus |
|  | 079.81 | Hantavirus infection |
|  | 079.82 | SARS-associated coronavirus infection |
|  | 382.0 | Acute suppurative otitis media |
|  | 382.4 | Unspecified suppurative otitis media |
|  | 382.9 | Unspecified otitis media |
|  | 460 | Nasopharyngitis, acute |
|  | 461 | Acute sinusitis |
|  | 462 | Pharyngitis, acute not otherwise specified |
|  | 463 | Acute tonsillitis |
|  | 464 | Acute laryngitis and tracheitis |
|  | 465 | Acute upper respiratory infections of multiple or unspecified sites |
|  | 466 | Acute bronchitis and bronchiolitis |
|  | 475 | Peritonsillar abscess |
|  | 480 | Viral pneumonia |
|  | 481 | Pneumococcal pneumonia |
|  | 482 | Other bacterial pneumonia |
|  | 483 | Pneumonia due to other specified organism |
|  | 484 | Pneumonia in infectious diseases classified elsewhere |
|  | 485 | Bronchopneumonia organism unspecified |
|  | 486 | Pneumonia, organism not otherwise specified |
|  | 487^a^ | Influenza |
|  | 488^a^ | Influenza due to identified avian influenza virus |
|  | 490 | Bronchitis not otherwise specified |
|  | 510 | Empyema |
|  | 511.1 | Pleurisy with effusion with a bacterial cause other than tuberculosis |
|  | 513.0 | Abscess lung |
| Gastrointestinal | 001 | Cholera |
|  | 002 | Typhoid and paratyphoid fevers |
|  | 003.0 | Other salmonella infections |
|  | 004 | Shigellosis |
|  | 005 | Other food poisoning (bacterial) |
|  | 006.0 | Acute amebic dysentery without abscess |
|  | 006.2 | Amebic nondysenteric colitis |
|  | 007 | Other protozoal intestinal diseases |
|  | 008 | Intestinal infections due to other organisms |
|  | 009 | Ill-defined intestinal infections |
|  | 078.82 | Epidemic vomiting syndrome |
| Unspecified viral infection | 079.99 | Unspecified viral infection |
| Diarrhea^b^ | 787.91 | Diarrhea |
| Fever^c^ | 780.6, 780.60, 780.61 | Fever and other physiologic disturbances of temperature regulation |
|  | Not applicable | Measured temperature of ≥100˚ Fahrenheit |

^a^Influenza was part of the general definition for respiratory tract infection, but was also considered separately in secondary analyses.

^b^Diarrhea was included only in secondary analyses for gastrointestinal infection, as it was considered non-specific for infectious etiology.

^c^Fever was included only in secondary analyses, to improve the specificity of the definition for upper or lower respiratory tract infection.
