# Supplementary material for: Guillain-Barré Syndrome, Influenza Vaccination, and Antecedent Respiratory and Gastrointestinal Infections: A Case-Centered Analysis in the Vaccine Safety Datalink, 2009–2011
Source: PLoS One. 2013 Jun 26;8(6):e67185. doi: 10.1371/journal.pone.0067185 (PMC3694016; doi:10.1371/journal.pone.0067185)
Supplement: Table S2 — Characteristics of confirmed Guillain-Barré syndrome (GBS) cases according to prior influenza vaccination and infection exposure status, Vaccine Safety Datalink, 2009–2011. (DOCX) [file pone.0067185.s002.docx]

**Table S2. Characteristics of confirmed Guillain-Barré syndrome (GBS) cases according to prior influenza vaccination^a^ and infection^b^ exposure status, Vaccine Safety Datalink, 2009-2011.**

| **Category** | **Level** | **Vaccinated and infected 1 through 42 days prior to GBS onset (n=3)** | **Vaccinated but not infected 1 through 42 days prior to GBS onset (n=15)** | **Infected but not vaccinated 1 through 42 days prior to GBS onset (n=41)** | **Vaccinated or infected 50 through 126 days prior to GBS onset (n=19)** | **Total (n=78)** |
| --- | --- | --- | --- | --- | --- | --- |
| Male | | 1 | 10 | 23 | 10 | 44 |
| Age group | ≤17 years | 0 | 4 | 7 | 2 | 13 |
|  | 18-49 years | 1 | 3 | 13 | 6 | 23 |
|  | 50-64 years | 2 | 4 | 10 | 4 | 20 |
|  | ≥65 years | 0 | 4 | 11 | 7 | 22 |
| Family history of GBS or FS | | 0 | 0 | 0 | 1 | 1 |
| Treated with intravenous immune globulin | | 3 | 12 | 38 | 17 | 70 |
| Received plasmapheresis | | 0 | 4 | 6 | 3 | 13 |
| Required intubation | | 1 | 1 | 9 | 1 | 12 |
| Died | | 0 | 1 | 0 | 0 | 1 |

^a^2009-10 monovalent inactivated influenza vaccine or 2010-11 trivalent inactivated influenza vaccine.

^b^Medically-attended respiratory tract, gastrointestinal, or unspecified viral infection.
