# Supplementary material for: Guillain-Barré Syndrome, Influenza Vaccination, and Antecedent Respiratory and Gastrointestinal Infections: A Case-Centered Analysis in the Vaccine Safety Datalink, 2009–2011
Source: PLoS One. 2013 Jun 26;8(6):e67185. doi: 10.1371/journal.pone.0067185 (PMC3694016; doi:10.1371/journal.pone.0067185)
Supplement: Table S3 — Numbers of patients with confirmed Guillain-Barré syndrome (GBS) by vaccination and infection exposure status and observation interval, Vaccine Safety Datalink, 2009–2011. (DOCX) [file pone.0067185.s003.docx]

**Table S3. Numbers of patients with confirmed Guillain-Barré syndrome (GBS) by vaccination^a^ and infection^b^ exposure status and observation interval, Vaccine Safety Datalink, 2009-2011.**

|  |  | **Infection** | | | |
| --- | --- | --- | --- | --- | --- |
|  |  | **None** | **1 through 42 days** | **50 through 126 days** | **Total** |
| **Influenza vaccination** | **None** | Not applicable^c^ | 36 | 6 | 42 |
|  | **1 through 42 days** | 13 | 3 | 2 | 18 |
|  | **50 through 126 days** | 13 | 5 | 0 | 18 |
|  | **Total** | 26 | 44 | 8 | 78 |

^a^2009-10 monovalent inactivated influenza vaccine or 2010-11 trivalent inactivated influenza vaccine.

^b^Medically-attended respiratory tract, gastrointestinal, or unspecified viral infection.

^c^GBS patients with neither prior exposure were ineligible for the study.
