# Supplementary material for: Guillain-Barré Syndrome, Influenza Vaccination, and Antecedent Respiratory and Gastrointestinal Infections: A Case-Centered Analysis in the Vaccine Safety Datalink, 2009–2011
Source: PLoS One. 2013 Jun 26;8(6):e67185. doi: 10.1371/journal.pone.0067185 (PMC3694016; doi:10.1371/journal.pone.0067185)
Supplement: Table S4 — Ascertainment of and adjustment for antecedent infection in selected prior observational studies of 2009–10 pandemic H1N1 vaccination and Guillain-Barré syndrome. (DOCX) [file pone.0067185.s004.docx]

**Table S4. Ascertainment of and adjustment for antecedent infection in selected prior observational studies of 2009-10 pandemic H1N1 vaccination and Guillain-Barré syndrome.**

| **Publication** | **Study design** | **Antecedent infection definition** | **Data source for antecedent infections** | **Results** | **Possible limitations regarding infection adjustment** |
| --- | --- | --- | --- | --- | --- |
| Liang et al., 2011 [41] | Passive surveillance comparing GBS rates in MIV vaccinees to baseline rates in the Chinese Acute Flaccid Paralysis  Surveillance System | History of infection | Passive reports from vaccinees | Eleven cases of GBS were reported, for a rate of 0.1 per 1 million doses, which was lower than the background rate in China. One of the 11 cases occurred in a patient with a history of infection and was therefore considered coincidental. | a, b |
| Dieleman et al., 2011 [42] | Case-control | Influenza-like illness/upper respiratory tract infection in prior six weeks | General practice records (UK and the Netherlands), hospital medical records (France), and structured interview (Sweden) | The increase in risk of GBS associated with pandemic influenza vaccination in unadjusted analyses disappeared upon adjustment through regression for influenza-like illness/upper respiratory tract infections and seasonal influenza vaccination (adjusted OR: 1.0, 95% CI: 0.3-2.7). | c |
| Grimaldi-Bensouda et al., 2011 [40] | Case-control | Report of influenza or influenza-like symptoms in prior two months | Patient telephone interview | GBS was not associated with influenza A (H1N1) vaccine (adjusted OR: 0.92, 95% CI: 0.11-7.55), but the point estimate of the association between GBS and infection was elevated (adjusted OR: 2.11, 95% CI: 0.57-7.73). | c |
| Andrews et al., 2011 [45] | Self-controlled case series | Preceding infection, including influenza | General practitioner reports | No pandemic vaccine recipients had an infection reported prior to onset. No association between GBS and pandemic influenza vaccine was identified (relative incidence: 1.05, 95% CI: 0.37-2.24). | d |
| Verity et al., 2011 [43] | Active surveillance comparing GBS rates in estimated person-time exposed vs. background rate from literature | Clinical and/or laboratory  evidence of an infection in  prior 3 months | Questionnaires to pediatricians who reported cases to the British Paediatric Surveillance Unit system | One GBS case was observed with onset within 6 weeks of H1N1 vaccination, consistent with the published background rate. Of 57 GBS/FS cases identified during the study period, 49 had an antecedent infection | d, e |
| Wise et al., 2012 [17] | Active surveillance comparing GBS rates in estimated person-time exposed vs. unexposed to MIV | Upper respiratory or  influenza-like symptoms, gastrointestinal symptoms in prior 6 weeks | Medical record review, patient telephone survey | The rate of GBS immediately following pH1N1 vaccine was 57% higher than in unexposed person-time (age- and sex-adjusted rate ratio = 1.57, 95% CI: 1.02-2.21). Antecedent upper respiratory or influenzalike symptoms were significantly less common among cases who received pH1N1 vaccine (38%) than among those who did not (67%). | d, e |
| Tokars et al., 2012 [16] | Self-controlled | Same as Wise et al. ^17^ | Same as Wise et al. ^17^ | A statistically significant association with GBS was found for MIV (RR: 2.1, 95% CI 1.2-3.5). No adjustments were made for infection. However, if seasonal infections affected the risk of GBS, the authors would have expected to see seasonality in GBS onset, but no such seasonality during 2009-10 was identified. | d, e, f, g |
| Greene et al., 2012 [15] | Self-controlled risk interval; case-centered | Upper respiratory infection and gastrointestinal illness in prior month | Medical record review | A statistically significant association with GBS was found for MIV (RR: 4.4, 95% CI: 1.3-14.2), but not TIV. Five of nine GBS cases with onset in the six weeks following MIV had an antecedent respiratory illness, compared with one of eight GBS cases following TIV. No GBS seasonality during 2009-10 was identified. | d, e, f, g |
| Yih et al., 2012 [18] | Self-controlled risk interval | Upper respiratory infection and gastrointestinal illness in prior 6 weeks | Medical record review | An elevated but not statistically significant association with GBS was found for MIV (2.50, 95% CI: 0.42-15.0). One of 3 cases with onset in the risk interval and 1 of 2 cases in the control interval had a respiratory or gastrointestinal illness within 6 weeks prior to GBS onset. | d, e, g |
| De Wals et al., 2012 [44] | Self-controlled case series | Respiratory tract infection, influenza-like illness, and gastroenteritis in prior month | Medical record review | In stratified SCCS analyses, the association between GBS and MIV was higher for patients with a negative history of respiratory tract infection or influenza-like illness (RR=4.1, 95% CI: 2.02-8.52) than for patients with a positive history (RR=1.24, 95% CI: 0.33-4.61). | d, e, h |

Abbreviations: CI (confidence interval); RR (relative risk); OR (odds ratio).

1. Passive reporting can yield unreliable information on relevant exposures, e.g., antecedent infection.
2. If a GBS patient had both antecedent vaccine and infection exposures, the cause of GBS was simply assumed to be the infection.
3. Case-control studies are subject to misclassification bias, i.e., differential recall or recording of antecedent infections between cases and controls. Appropriate controls would need to have a non-GBS condition for which they would be as likely to recall or to be asked about an antecedent infection as a GBS patient. Otherwise, antecedent infections may be systematically under-ascertained in medical records of control patients.
4. Data regarding antecedent infections were obtained only for vaccination-exposed person-time for cases (rather than also for vaccination-unexposed person-time), so it was not possible to formally adjust for infection in the analyses.
5. Because exposure information is recorded in the chart *after* GBS onset, medical records are subject to information bias. Patients with a possible alternative documented cause of GBS (such as vaccination) may not have other relevant exposures adequately recorded (such as infection).
6. It is unclear whether there is general seasonality in GBS incidence.^33^ A prior study in the VSD population reported significantly higher GBS rates in winter and spring compared with other seasons.^5^ A lack of demonstration of seasonality for a rare event in one year from one data source does not prove that seasonal exposures such as vaccination and infection are not important, as a robust seasonality assessment may be infeasible using a self-controlled design.
7. 2009-10 MIV administration and wild-type infections coincided, such that some cases in the risk interval following vaccination may have been due to infection, while cases in the control interval following vaccination were unlikely to be due to infection.
8. It is not valid to use a self-controlled case series design restricting to a subpopulation of patients with a positive history of infection relative to GBS, without adjusting for the time-varying risk of that infection. For each case, if the timing of the infection was in the risk interval for vaccination, some of the risk in the vaccination risk interval may be due to infection, but all elevated risk is attributed to the vaccine, biasing away from the null. In contrast, if the timing of the infection was in the control interval for vaccination, then the control interval is an elevated estimate of the baseline risk, biasing toward the null.
